# Evaluation of Forensic Data Using Logistic Regression-Based Classification Methods and an R Shiny Implementation

**DOI:** 10.3389/fchem.2020.00738

**Published:** 2020-10-21

**Authors:** Giulia Biosa, Diana Giurghita, Eugenio Alladio, Marco Vincenti, Tereza Neocleous

**Affiliations:** ^1^Forensic Toxicology Laboratory, Department of Health Surveillance and Bioethics, Catholic University of the Sacred Heart, F. Policlinico Gemelli IRCCS, Rome, Italy; ^2^School of Mathematics and Statistics, University of Glasgow, Glasgow, United Kingdom; ^3^Forensic Biology Unit, Carabinieri Scientific Investigations Department of Rome, Rome, Italy; ^4^Department of Chemistry, University of Turin, Turin, Italy; ^5^Anti-doping and Toxicology Center “A. Bertinaria” of Orbassano, Turin, Italy

**Keywords:** classification, likelihood ratio, logistic regression, separation, forensic science, C_llr_

## Abstract

We demonstrate the use of classification methods that are well-suited for forensic toxicology applications. The methods are based on penalized logistic regression, can be employed when separation occurs in a two-class classification setting, and allow for the calculation of likelihood ratios. A case study of this framework is demonstrated on alcohol biomarker data for classifying chronic alcohol drinkers. The approach can be extended to applications in the fields of analytical and forensic chemistry, where it is a common feature to have a large number of biomarkers, and allows for flexibility in model assumptions such as multivariate normality. While some penalized regression methods have been introduced previously in forensic applications, our study is meant to encourage practitioners to use these powerful methods more widely. As such, based upon our proof-of-concept studies, we also introduce an R Shiny online tool with an intuitive interface able to perform several classification methods. We anticipate that this open-source and free-of-charge application will provide a powerful and dynamic tool to infer the LR value in case of classification tasks.

## 1. Introduction

A fundamental task for the forensic experts is that the results of the analyses, which have been performed on the collected pieces of evidence, have to be expressed in a very clear and straightforward way that can be easily shown in courtrooms and that can be immediately, where possible, understood even by non-specialists. However, at the same time, the applied statistical methodologies for data evaluation have to be rigorous and should not compromise the role that the forensic expert plays in the administration of justice (Zadora, 2010).

The main aim of the forensic analysts is to evaluate the physicochemical data from the collected evidence (*E*) in the framework of two independent, alternative, and mutually exclusive hypotheses (or propositions), *H*_1_ and *H*_2_, in order to estimate the conditional probabilities (*P*) related to the mentioned hypotheses [i.e., *P*(*E*|*H*_1_) and *P*(*E*|*H*_1_), which stand for the probability to observe the results from *E* when *H*_1_ (or *H*_2_) is true]. The comparison between the described conditional probabilities is performed by their ratio, and it is known as likelihood ratio (LR):

(1) $$\begin{array}{l} LR=\frac{P\left(E|H_{1}\right)}{P\left(E|H_{2}\right)} \end{array}$$

The LR has been largely adopted in forensic sciences (and courtrooms too) in recent years since it expresses the strength of the observed evidence in favor of proposition *H*_1_ compared to proposition *H*_2_ in a very straightforward way. It can be calculated on both discrete and continuous data, and it is not an assignment of a probability but rather a ratio of probabilities (or density functions for continuous data) and takes values from 0 to +∞. The value of LR equal to 1 represents the condition where the probability of observing the collected evidence when *H*_1_ is true is equal to the probability of *E* when *H*_2_ is true. In this case, the LR is inconclusive since it provides no support to either of the evaluated propositions. Conversely, the higher the value of the LR, the stronger the support for *H*_1_; on the other hand, the lower the value of the LR, the stronger the support for *H*_2_.

Furthermore, one of the most appreciated features of the LR is that it can be immediately converted into a statement by using well-known verbal scales (Evett et al., 2000). Nowadays, one of the most used scales is the one provided by the European Network of Forensic Sciences Institute (ENFSI) (European Network of Forensic Science Institutes, 2016), which is as follows: for 1 < *LR* ⩽ 10^1^, there is weak support for *H*_1_ rather than the alternative *H*_2_, for 10^1^ < *LR* ⩽ 10^2^ moderate support, for 10^2^ < *LR* ⩽ 10^3^ moderately strong support, for 10^3^ < *LR* ⩽ 10^4^ strong support, for 10^4^ < *LR* ⩽ 10^5^ very strong support, and for *LR* > 10^5^ extremely strong support. The same approach is used for LR values lower than 1.

Another relevant feature of applying the LR in the field of forensics is that it overcomes the so-called “falling off a cliff” problem related to the traditional approach of using cut-off values in classification models (Gill et al., 2006; Pragst et al., 2010; Zadora, 2010; Robertson et al., 2016). In particular, the use of LR avoids the necessity of defining thresholds (such as the largely adopted *p*-value = 0.05 for a significance level of 95%). As a matter of fact, the use of a cut-off such as *p* = 0.05 leads the forensic analyst to completely different (and opposite) conclusions when values close to the defined threshold are observed, such as the cases of *p* = 0.049 or *p* = 0.051. For the first scenario, the proposition *H*_1_ can be rejected, while, for the second scenario, *H*_1_ cannot be rejected. Furthermore, no conclusion can be made about the alternative proposition *H*_2_ because *H*_2_ is not taken into account for the calculation of the *p*-value. Moreover, a very small difference in the calculated *p*-value produces a completely different interpretation of the obtained results, thus leading to possible severe consequences for the subjects under investigation (Wasserstein and Lazar, 2016). This approach is particularly inadvisable when dealing with multivariate data since small differences in the calculated probability values (such as, for instance, *p*-values) could be ascribed to really small differences in the performed analytical measurements. This problem does not occur when the likelihood ratio approach is adopted since the LR does not require the definition of a threshold. Moreover, the degree of support to be delivered to a certain proposition rather than its alternative can be easily related to the magnitude of the LR through the expressions of the cited verbal equivalents.

For these reasons, LR approaches have been largely used in many applications of forensic sciences (Aitken and Taroni, 2004) starting from DNA profiling (Evett and Weir, 1998; Gill et al., 2006) to other forensic fields such as the evaluation of fire debris (Zadora et al., 2005), car paints (Martyna et al., 2015; Michalska et al., 2015), glass fragments (Zadora and Ramos, 2010; Zadora et al., 2014), speaker recognition (Ramos, 2007), and forensic voice comparison with Gonzalez-Rodriguez et al. (2007) being one of the earliest works to introduce LRs derived from logistic regression in the latter field. Another investigated field for the application of LR is the discrimination between chronic and non-chronic alcohol drinkers (Alladio et al., 2017a,b, 2018).

According to the World Health Organization, excessive alcohol consumption is a causal factor in more than 200 disease and injury conditions. Furthermore, the abuse of alcohol severely influences the consumers' lives, leading to different legal, physical, and psychological consequences, especially when dealing with behaviors that might cause road and work accidents.

In recent decades, great interest has been dedicated to the identification of biomarkers capable of recognizing individuals with alcohol-related problems, for both prevention and monitoring purposes. The current approach, from a toxicological point-of-view, aims to identify a person who falls into the category of excessive alcohol consumer through the analysis of indirect biomarkers of alcohol consumption (in blood/serum samples) and, mainly, direct biomarkers (in hair samples, with a length of 0–6 cm) (Kintz et al., 2015).

The most frequently analyzed indirect biomarkers, whose concentration levels in blood are not directly related to the alcohol consumption since they are not formed by alcohol metabolic processes, are aspartate transferase (AST), alanine transferase (ALT), gamma-glutamyl transferase (GGT), mean corpuscular volume of the erythrocytes (MCV), and carbohydrate-deficient-transferrin (CDT) (Pirro et al., 2013). These biomarkers are less frequently evaluated nowadays since they significantly disclose the harmful effects of alcohol on target organs but provide unsatisfactory results in terms of sensitivity and specificity. On the other hand, ethyl glucuronide (EtG) and fatty acid ethyl esters (FAEEs) are the most widely evaluated direct biomarkers of alcohol consumption in hair samples. In particular, EtG is used as reference biomarker since it shows admirable diagnostic sensitivity and specificity results, being capable of assessing both chronic alcohol drinkers (with a cut-off of 30 pg/mg) and teetotaller individuals (with a cut-off of 5 pg/mg). These values have been defined by the Society of Hair Testings (SoHT) and accepted by the forensic community (Society of Hair Testing, 2019).

On the other hand, the determination of FAEEs in hair samples is performed to assist the decision process on chronic alcohol abuse by adding a second biomarker that can be exploited in case of doubtful circumstances (e.g., in case of EtG values close to the 30 pg/mg cut-off). In particular, FAEEs are a family of more than twenty compounds that are synthesized by non-oxidative metabolic esterification processes of fatty acids following the drinking of alcohol. Traditionally, four most present FAEEs were quantified [i.e., ethyl myristate (E14:0), ethyl palmitate (E16:0), ethyl oleate (E18:0), and ethyl stearate (E18:1)] and their sum was calculated. Moreover, recently, ethyl palmitate (E16:0) has been proposed for alternative interpretation, instead of the sum of the four FAEEs, and possible cut-off values for E16:0 have been updated by the Society of Hair Testing (SoHT), i.e., 0.35 ng/mg for 0–3 cm proximal hair segment and 0.45 ng/mg for 0–6 cm proximal hair segment (Society of Hair Testing, 2019).

The dataset featured in this paper (described in section 2.8) includes samples of both direct and indirect biomarkers of ethanol consumption, collected from two types of alcohol drinkers: chronic and non-chronic. Given the multivariate nature of the data, several multivariate data analysis methods have been proposed to analyze this dataset: Linear Discriminant Analysis (LDA) (Mai, 2013), Quadratic Discriminant Analysis (QDA) (Qin, 2018), binary logistic regression (Murphy, 2012, Chapter 8), as well as penalized versions of logistic regression. The last class of methods, comprising of Firth generalized linear model (Firth GLM) (Firth, 1993), Bayes generalized linear model (Bayes GLM) (Gelman et al., 2008), and GLM-NET (Friedman et al., 2001), have been included to deal with data separation, which occurs when the class variable is perfectly separated by one or more measurement variables. A description of these methods is presented in section 2, while in section 3, we demonstrate how to identify separation in a dataset and discuss the results of a comparison study with the aforementioned methods.

Lastly, in section 3.2, we introduce an R Shiny app, which has been originally developed to provide forensic experts and physicians with a straightforward tool capable of discriminating chronic alcohol drinkers from non-chronic alcohol drinkers through the combination of multivariate data analysis techniques and LR models (involving both uni- and multivariate approaches) following the approaches and the results reported in Alladio et al. (2017b). The R Shiny app can be used with any classification datasets, from any area of forensic science, and for clinical and toxicological purposes. All the methods discussed in this paper are implemented and can be used for obtaining LRs and class prediction. The app has been tested on several well-known datasets that are commonly used in machine learning for classification, such as iris (Dua and Graff, 2019), glass (Dua and Graff, 2019), Pima diabetes (Dua and Graff, 2019), and diamonds (Wickham, 2016), and these are also available to users as part of the R Shiny app to demonstrate its capabilities.

Although the ideas of using penalized (or regularized) logistic regression and kernel density estimation have been previously explored in the context of score-based forensic analysis in applications such as glass (Morrison and Poh, 2018) and voice comparison (Morrison, 2011a), to our knowledge, the particular methods explored in this paper and the R Shiny tool we provide are relatively novel to the forensic sciences.

## 2. Materials and Methods

In this section, we describe logistic regression-based classification methods and we introduce the ideas behind various penalized logistic regression approaches, indicating how they can extend existing methodology commonly used with forensic data. Furthermore, we provide an overview of classification performance measures used to assess the goodness of fit for classification models, and we provide some ideas for how to design a comparison study between several candidate models using cross-validation. Lastly, we provide a description of the alcohol biomarkers dataset (Alladio et al., 2017a) used to illustrate these methods.

In statistics, classification methods tackle the problem of determining the category, or class membership, of an object based on a set of explanatory variables, or features, describing it. The methods covered in this paper are referred to as “supervised learning,” meaning a set of observations is typically available whose class membership is *known*. These observations form a training set which is used by a statistical algorithm, or classifier, to map the features of the object to its class through mathematical functions. Ultimately, the purpose of the classifier is to apply the mathematical functions obtained using the training set to determine the class of new objects, based on the same features recorded in the training set. An obvious example of classification is medical diagnostics, where a doctor has to assign a diagnostic to a patient based on different variables: sex, age, blood pressure, etc. In the context of monitoring chronic alcohol abuse, it is of interest to perform classification into one of two categories: chronic or non-chronic alcohol drinker.

### 2.1. Linear and Quadratic Discriminant Analysis

Linear Discriminant Analysis (LDA) and Quadratic Discriminant Analysis (QDA) are two widely used methods for classification, which, as suggested by their names, produce a linear or quadratic decision boundary between classes. These algorithms can be easily computed and have simple mathematical formulations, are suitable for binary and multi-class classification problems and have no additional parameters that need to be tuned.

A discriminant function is a mathematical function that maps each observation's features directly to a specific class. In the case of LDA, the algorithm estimates weights for each of the features such that the estimated classification is a linear combination of an observation's features and the separation between class means is maximized, while the spread within each class is minimized (Bishop, 2006, Chapter 4).

It should be mentioned that LDA assumes normally distributed (Gaussian) data, features that are statistically independent, and identical covariance matrices for every class. QDA is a more general algorithm for which the assumption of identical covariance matrices is not necessary, and the result is, in essence, a more flexible decision boundary. However, if the distribution of the data is significantly non-Gaussian, then it is very likely that neither LDA nor QDA will perform very well.

### 2.2. Logistic Regression

A logistic regression model is typically used to identify the relationship between independent variables *X*_*i*_ and a response or dependent variable *Y* that is binary, meaning it can take two values: e.g., True or False, 1 or 0, chronic or non-chronic drinker, etc. For illustrative purposes and without loss of generality, we assume the *Y* variable is labeled as positive (Category 1) or negative (Category 2) for a dataset with *N* observations. The mathematical form of a logistic regression model is described in Equation (2):

(2) $$\begin{array}{l} \text{logit}\left(p\right)=\log\left(\frac{p}{1-p}\right)=\beta_{0}+\beta_{1}X_{1}+\ldots +\beta_{k}X_{k} \end{array}$$

where β_1_, …, β_*k*_ are models parameters that need to be estimated, *k* is the number of independent variables, and *p* is the probability of success: *P*(*Y* = positive) = *p*. Once the parameters have been estimated, the logistic regression model equation allows us to calculate probabilities for each class of the response variable (Equation 3), as well as the odds (Equation 4), which denotes the ratio of the probability of success to the probability of failure.

The probability is expressed as a function of the predictors in terms of the logistic equation:

(3) $$\begin{array}{l} p=\frac{\exp\left(\beta_{0}+\beta_{1}X_{1}+\ldots +\beta_{k}X_{k}\right)}{1+\exp\left(\beta_{0}+\beta_{1}X_{1}+\ldots +\beta_{k}X_{k}\right)} \end{array}$$

and the odds as

(4) $$\begin{array}{l} \frac{p}{1-p}=\exp\left(\beta_{0}+\beta_{1}X_{1}+\ldots +\beta_{k}X_{k}\right). \end{array}$$

The odds is a ratio of probabilities, and if it is greater than 1 (if *p* > 0.5, then *p*/(1 − *p*) > 1) we classify it as Category 1 (positive), while if it is smaller than 1 (if *p* < 0.5, then *p*/(1 − *p*) < 1) we classify it as Category 2 (negative). This can be compared with the use of the LR for classification.

In the case of a classification problem with two mutually exclusive and exhaustive categories, there is a parallel between *p* and *P*(*H*_1_|*E*) and between 1 − *p* and *P*(*H*_2_|*E*) where *H*_1_, *H*_2_, and *E* were defined in section 1. Writing the posterior odds as prior odds times the likelihood ratio gives

(5) $$\begin{array}{l} \frac{P\left(H_{1}|E\right)}{P\left(H_{2}|E\right)}=\frac{P\left(H_{1}\right)}{P\left(H_{2}\right)}\cdot \frac{P\left(E|H_{1}\right)}{P\left(E|H_{2}\right)} \end{array}$$

providing a relationship between the likelihood ratio, $\frac{P\left(E|H_{1}\right)}{P\left(E|H_{2}\right)}$, and the probability *p* in Equation (2). For training the logistic regression models used in the remainder of this paper, the prior odds $\frac{P\left(H_{1}\right)}{P\left(H_{2}\right)}$ was assumed to take the value 1, in which case the LR equals $\frac{p}{1-p}$. For different values of the prior odds, $\log\frac{P\left(H_{1}|E\right)}{P\left(H_{2}|E\right)}$ can be obtained from the model described in Equation (2) assuming a fixed value of the prior log-odds $\log\frac{P\left(H_{1}\right)}{P\left(H_{2}\right)}$. The value of the log(LR) can then be calculated as $\log\frac{P\left(H_{1}|E\right)}{P\left(H_{2}|E\right)}-\log\frac{P\left(H_{1}\right)}{P\left(H_{2}\right)}$.

For logistic regression, estimation problems can arise when dealing with a large number of variables relative to the number of observations (*N* close to “*k*” scenario) or when perfect or quasi-separation occurs (Heinze and Schemper, 2002; Gelman et al., 2008). The latter scenario refers to situations when one explanatory variable or a combination of explanatory variables completely separate the classes in the dataset. Separation will normally be flagged up by the algorithm implementation, but this can also be observed by inspecting the estimated coefficients and standard errors, which will typically be very large, or by looking at the estimated LRs, which will typically be infinite or zero and is obviously unrealistic. In a logistic regression model, the standard errors associated with each coefficient give a measure of the uncertainty about the coefficient estimate and can be used to test whether the coefficients are significantly different from 0 or to construct confidence intervals for each predictor added in the model. One indication of separation in a logistic regression model is when the estimated standard errors are orders of magnitude larger than the value of the coefficients.

One way to address this problem is to fit a penalized or Bayesian logistic regression model, for example, GLM-NET, Firth GLM, or Bayes GLM, all of which are briefly described in rest of this section.

### 2.3. GLM-NET

GLM-NET described in Friedman et al. (2010) and implemented in R package glmnet (Hastie and Qian, 2014), comprises of fast algorithms that estimate generalized linear models with convex penalties, such as ℓ_1_ (the lasso), ℓ_2_ (ridge regression) and mixtures of these two, generally known as elastic net penalties.

Ridge regression is a method commonly used when dealing with a large number of explanatory variables, which will inevitably be correlated. The ridge penalty allows many predictors to be included in a model by shrinking the corresponding coefficients of correlated variables toward each other or shrinking less important variable coefficients toward 0. It is important to note that all resulting regression coefficients will be non-zero. On the other hand, lasso will pick one of the correlated variables and ignore the rest, while the elastic-net penalty mixes these two behaviors (regularizes but also excludes variables) (Friedman et al., 2010).

Glmnet is a package that fits a generalized linear model via penalized maximum likelihood. The algorithm is very fast, using cyclical coordinate descent—it successively optimizes the objective function for each parameter in turn while the others are kept fixed until convergence is achieved. It can be used with a variety of models: linear, logistic, and multinomial, Poisson, and Cox regression models and can also accommodate multi-class scenarios.

GLM-NET performs regularized logistic regression by maximizing the following penalized log likelihood:

(6) $$\begin{array}{l} \underset{(\beta_{0},\beta )\in {\mathbb{R}}^{k+1}}{\text{max}}[\frac{1}{N}\sum_{i=1}^{N}\left[Y_{i}(\beta_{0}+X_{i}^{T}\beta )-\text{log}\left(1+e^{\left(\beta_{0}+X_{i}^{T}\beta \right)}\right)\right]\\ -\lambda P_{\alpha }(\beta )] \end{array}$$

where

(7) $$\begin{array}{l} P_{\alpha }\left(\beta \right)=\left(1-\alpha \right)\frac{1}{2}||\beta |{|}_{\ell_{2}}^{2}+\alpha ||\beta |{|}_{\ell_{1}} \end{array}$$

(8) $$\begin{array}{l} =\overset{k}{\underset{j=1}{\sum }}\left[\frac{1}{2}\left(1-\alpha \right)\beta_{j}^{2}+\alpha |\beta_{j}|\right] \end{array}$$

*P*_α_ is the elastic-net penalty containing the ridge and lasso penalties as special cases when α = 0 and α = 1, respectively. λ represents a tuning parameter that controls the overall strength of the penalty and can be chosen using cross-validation within the glmnet package.

### 2.4. Firth GLM

Firth GLM described in Heinze and Schemper (2002) and Firth (1993), implemented in R package brglm2 (Kosmidis, 2020), proposes a solution to the separation problem that involves maximizing a log likelihood penalized by Jeffreys prior (Firth, 1993):

(9) $$\begin{array}{l} \underset{\beta \in {\mathbb{R}}^{k+1}}{\max}\overset{N}{\underset{i=1}{\sum }}\left[Y_{i}\log\left(p_{i}\right)+\left(1-Y_{i}\right)\log\left(1-p_{i}\right)\right]+\frac{1}{2}I\left(\beta \right) \end{array}$$

where *I*(β) denotes the Fisher information matrix evaluated at β.

This penalty effectively removes bias from parameter estimates which can be quite serious in sparse or small datasets. In the case of separated data, the profile penalized likelihood is used to construct confidence intervals, see Heinze and Schemper (2002) and Kosmidis et al. (2020) for a more in-depth discussion.

### 2.5. Bayes GLM

Bayes-GLM described in Gelman et al. (2008) and Chapter 16 of Gelman et al. (2013), implemented in R package arm (Gelman et al., 2018), provides a fully Bayesian formulation of a logistic regression model, using weakly informative priors such as Student-*t* or Cauchy prior distributions for the regression coefficients.

Point estimates and standard errors for the regression coefficients are obtained using a pseudo-data approach, which computes estimates by a modified iteratively weighted least squares algorithm, using a prior-augmented design matrix, *X*, vector of observations, and weights vector. Section 16.3 in Gelman et al. (2013) provides an extensive illustration of the Bayes-GLM approach.

According to the authors, the choice of weakly informative priors provides regularization and stabilization to the algorithm which are superior to other similar methods such as Firth logistic regression, where the choice of prior (Jeffreys) does not ensure a stable enough estimation.

### 2.6. Combining LRs

In addition to its use as a classification tool for a two-category problem, as described in section 2.2, logistic regression can be used to produce a weighted average of LRs (called “scores”) obtained using different pieces of evidence (or variables). This approach, sometimes referred to as logistic regression fusion, is routinely used in forensic voice comparison and is described in detail in Morrison (2013). This method is especially useful for multivariate data when the number of variables is relatively large compared to the number of observations.

In general, the fusion model can be written as follows:

(10) $$\begin{array}{l} \text{logit}\left(p\right)=\log\left(\frac{p}{1-p}\right)=\beta_{0}^{*}+\beta_{1}^{*}s_{1}+\ldots +\beta_{k}^{*}s_{k} \end{array}$$

where $\beta_{0}^{*},\beta_{1}^{*},\ldots ,\beta_{k}^{*}$, are the logistic regression coefficients that have to be estimated, and $\frac{p}{1-p}$ is the LR of interest for the practitioner (assuming prior odds of 1, as in section 2.2). In this model *s*_1_, *s*_2_, …, *s*_*k*_ are scores, typically obtained from different data variables or measurements available in the dataset. These scores can be derived as follows: first, we estimate the distribution of both classes for each of the variables of interest, and then we calculate the LR (ratio of the probabilities) for a specific measurement *x* under each class distribution:

(11) $$\begin{array}{l} s_{i}=\log\left(LR\right)=\log\frac{f\left(x|H_{1}\right)}{f\left(x|H_{2}\right)} \end{array}$$

**Figure 1:**
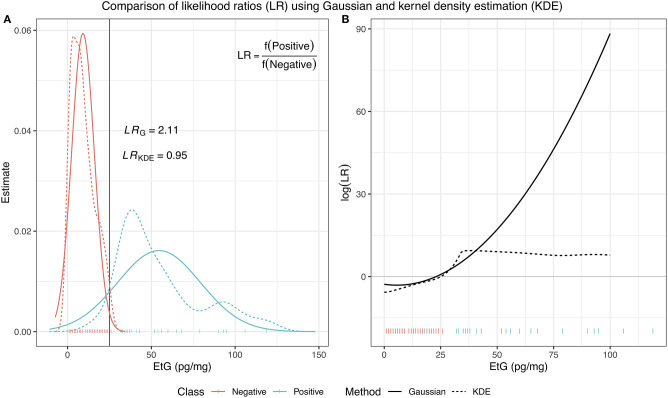
A comparison of LRs using two different methods of density estimation: a Gaussian distribution and kernel density estimator. In **(A)**, for an EtG value of 25, the Gaussian LR has a value of 2.11, indicating the class of an observation with this EtG value should be positive, while the KDE LR has a value of 0.95, indicating that the observation provides similar support for membership of either class. **(B)** Shows the resulting log(LR) evaluated for observations with EtG values between 0 and 100 and illustrates how different the results can be depending on the choice of estimation for the underlying distribution of the data.

In Equation (11), the function *f* represents the density estimates of each class, which is estimated from a training dataset, where the class membership for each observation is known. Most commonly, these two distributions are assumed to be Gaussian, meaning $f\left(x\right)=\frac{1}{\sigma \sqrt{2\pi }}\exp\frac{{\left(x-\mu \right)}^{2}}{-2\sigma^{2}}$, however, this is not always suitable. In such situations, kernel density estimation (KDE) can be used specifically to deal with issues such as estimating a multimodal distributions or an unusual behavior in the tails, which the Gaussian distribution can often struggle with. In Figure 1, we illustrate these two methods of density estimation and how different the resulting log(LR) can be based on the chosen method.

Intuitively, KDE estimates the underlying density function of some samples *x*_1_, …, *x*_*n*_ by placing a Gaussian distribution over each data point, adding the contributions from each point, and dividing by *n* to ensure the resulting function is normalized. The Gaussian can be replaced by other non-negative functions, or kernels, denoted *K*, in which case the resulting density estimate, $\hat{f}$ can be written as follows:

(12) $$\begin{array}{l} f\left(x\right)\approx \hat{f}\left(x\right)=\frac{1}{nh}\overset{n}{\underset{i=1}{\sum }}K\left(\frac{x-x_{i}}{h}\right) \end{array}$$

In Equation (12), *h* represents the bandwidth, a parameter which determines how smooth or spiky the resulting density estimation curve should be. In this paper, we use a Gaussian kernel with a bandwidth parameter set according to Silverman's rule (Silverman, 1986, p. 48):

(13) $$\begin{array}{l} h=0.9\cdot \min\left(\hat{\sigma },\frac{IQR}{1.34}\right)\cdot n^{-0.2} \end{array}$$

where $\hat{\sigma }$ and IQR are the standard deviation and interquartile range of the *x*_*i*_'s. The bandwidth parameter can also be chosen using other methods, such as cross-validation, however this comes at an increased computational cost. More discussion on kernel density estimators and the choice of bandwidth can be found in Bishop (2006, Chapter 2.5).

Once the scores are obtained from the variables of interest, the next stage is to use the logistic regression fusion model in Equation (10) to combine all the available pieces of evidence to obtain a calibrated LR. Here, the estimated logistic regression coefficients, $\beta_{1}^{*},\ldots ,\beta_{k}^{*}$ can be regarded as weights for each score or variable included in the model, providing an extra bonus of ease of interpretation. Since the fusion approach involves a two-stage estimation procedure, to avoid overfitting (obtaining overly optimistic estimates), we use two different datasets at each of these stages, a training dataset to estimate the class distribution for each explanatory variable, and a validation dataset to estimate the logistic regression fusion model coefficients.

In this paper, the fusion method is applied using each of the penalized logistic regression models introduced previously: GLM-NET, Firth GLM, and Bayes GLM, as these methods are equipped to deal with correlated variables and separation issues, both of which can happen the more variables are added to a model.

### 2.7. Classification Performance Measures

This section presents the classification measures used to assess the performance of the methods presented in this paper.

The first type of measures rely on the initial calculation of the confusion matrix—a table that allows the visualization of an algorithm's performance based on the numbers of observations correctly and incorrectly classified for each class. An example confusion matrix is provided in Table 1, based on the correctly and incorrectly classified observations, denoted as follows:

- true positives (TP): outcomes where the model correctly predicts a positive class.
- true negatives (TN): outcomes where the model correctly predicts a negative class.
- false positives (FP): outcomes where the model incorrectly predicts a positive class.
- false negatives (FN): outcomes where the model incorrectly predicts a negative class.

**Table 1:** An example of a confusion matrix. *N*_+_ is the number of true positives, ${\hat{N}}_{+}$ is the number of estimated positives, *N*_−_ is the number of true negatives, and ${\hat{N}}_{-}$ is the number of estimated negatives.

		**Truth**		
		**1**	**0**	**∑**
Estimate	1	*TP*	*FP*	${\hat{N}}_{+}=TP+FP$
	0	*FN*	*TN*	${\hat{N}}_{-}=FN+TN$
	∑	*N*_+_ = *TP*+*FN*	*N*_−_ = *FP*+*TN*	*N* = *TP*+*FP*+*FN*+*TN*

The classification for each observation is based on the choice of a threshold for the probability estimated by a model. Changing this threshold will change the values of TP, TN, FP, and FN and, consequently, all the measures calculated based on these. Throughout this paper, we use a threshold of 0.5 for the estimated probabilities (corresponding to a threshold of 1 in terms of the LR), meaning an observation with an estimated probability above 0.5 gets classified as a positive and an observation below 0.5 gets classified as a negative.

Table 2 shows a list and definitions of the following classification performance measures including those based on the confusion matrix: precision, recall, specificity, accuracy, error, and F1. Precision and recall are widely used to assess the number of false positives and false negatives, respectively. However, depending of the application, other measures might be of interest, such as the proportion of observations correctly classified (accuracy), the proportion of observations misclassified (error), or the proportion of negatives that are correctly identified (specificity). Often, it is difficult to compare two models with low precision and high recall or vice versa. To make them comparable, the F1 score, which is the harmonic mean of the two, is often used in practice. Murphy (2012, p. 181) presents a more in-depth discussion of classification measures.

**Table 2:** Classification performance measures and corresponding formulae and descriptions for metrics based on the confusion matrix and the C_*llr*_, a log likelihood ratio cost metric defined in Equation (14).

**Measure**	**Formula**			**Description**
Precision	$\frac{TP}{{\hat{N}}_{+}}$			Measures what proportion of the detected values are actually positive. A model that produces no false positives has a precision of 1.
Recall	$\frac{TP}{N_{+}}$			Also called *true positive rate* or *sensitivity*. It measures what proportion of the positives were actually detected. A model that produces no false negatives has a recall of 1.
Specificity	$\frac{TN}{N_{-}}$			Also called *true negative rate*. Measures the proportion of negatives that are correctly identified by the model.
Accuracy	$\frac{TN+TP}{N}$			Rate of correctly classified observations—how many observations were detected correctly out of both classes combined. Best value is 1, worst value is 0.
Error	$\frac{FP+FN}{N}$			Rate of incorrectly classified observations (*classification error*)—how many observations (either positive or negative) were incorrectly classified. Best value is 0, worst value is 1.
F1	$\frac{2\cdot Recall\cdot Precision}{Recall+Precision}$			The harmonic mean of the precision and recall measures, and it is often used in cases with low prevalence by penalizing extreme values. Best value is 1, worst value is 0.
C_llr_	see Equation (14)			Log likelihood ratio cost. Lower values indicate better performance.

Morrison (2011b) explains the downside of using measures such as classification accuracy and error by pointing to the fact that thresholding probabilities to assign class membership to an object lose important information regarding the strength of the evidence in favor of each class. By working directly with the LRs corresponding to each class, one can preserve the strength of evidence in favor of each proposition. As such, we include another classification performance indicator based on the log likelihood ratio cost, which was proposed in the forensic sciences—the C_llr_ (Morrison, 2011b; Ramos et al., 2018). This metric is implemented in the R package comparison (Lucy, 2013).

The formula for C_llr_ is as follows:

(14) $$\begin{array}{l} {\text{C}}_{\text{llr}}=\frac{1}{2}\left(\frac{1}{N_{1}}\overset{N_{1}}{\underset{i=1}{\sum }}\log\left(1+\frac{1}{LR_{1i}}\right)+\frac{1}{N_{2}}\overset{N_{2}}{\underset{j=1}{\sum }}\log\left(1+LR_{2j}\right)\right) \end{array}$$

where *N*_1_ and *N*_2_ represent the number of positive (Category 1) and negative (Category 2) sample comparisons, respectively, and *LR*_1_ and *LR*_2_ are the likelihood ratios derived from test pairs known to be of positive and negative origin, respectively.

#### 2.7.1. Cross-Validation

While in the previous section we described various classifiers and presented some measures of tracking how good a classifier is, in this section we describe how to design a comparison study between multiple algorithms using cross-validation. Cross-validation can be employed to avoid biased estimates and overfitting issues due to training and testing a classifier on the same dataset. In practice, the most common problems are either the lack of future data (for which the label has to be predicted) or small sample size datasets, which one would ideally use to their fullest capacity to train a classifier (Murphy, 2012).

Cross-validation provides an answer to this problem by partitioning data into a *training set*, which is used for fitting the models, and a *test set*, which is used for predicting the class labels and obtaining the performance measures (or prediction error, or any sort of indicator of goodness-of-fit for the models). The partitioning of the dataset is repeated in all possible ways, making sure that the same observation is not included for testing and training at the same time. Ultimately, the cross-validation goal is to obtain an out-of-sample prediction error for each algorithm, and this is done by averaging the classification error or measures considered over all repetitions. More details and discussion about cross-validation can be found in Venables and Ripley (2013), Murphy (2012), and Gelman et al. (2013).

One of the downsides of cross-validation is that it can get computationally expensive depending on the size of the dataset and the number of models included in the comparison, and a few variations of this procedure have been proposed to address this. Commonly used cross-validation procedures include exhaustive and non-exhaustive methods, based on whether they use all possible ways to split the dataset into training and testing sets or not. It should be noted that, in the latter case, the average score produced is an *approximate* cross-validation score.

Examples of commonly used cross-validation procedures include:

- *leave-p-out cross-validation*: *p* observations are used as a testing set and the remaining as the training set. This procedure is repeated in all possible ways of choosing *p* observations out of the total sample size, and, as expected, becomes unfeasible for even moderately large datasets (there are $C_{p}^{n}$ ways to choose *p* observations from a dataset of size *n*).
- *k-fold cross-validation*: the dataset is partitioned into *k* subsets of equal size, one of them being used for testing the algorithms, while the other *k*−1 being used for training. This is repeated such that each fold, and thus each observation, is used once for testing the models.

In this paper, we use a slightly different cross-validation procedure, due to the fact that some methods investigated (combining LRs using logistic regression fusion) involve multi-step estimation procedure and require the estimation of additional parameters. These parameters should ideally be estimated using data that is neither part of the training, nor testing sets—a further split, forming a *validation* set can be used in this scenario.

### 2.8. Data

The alcohol biomarker dataset published in Alladio et al. (2017a) presents concentration values of a series of direct and indirect biomarkers of ethanol consumption from 125 individuals classified as either chronic (positive) of non-chronic (negative) alcohol drinkers.

Indirect biomarkers, collected from blood, are as follows: aspartate transferase (AST), alanine transferase (ALT), gamma-glutamyl transferase (GGT), mean corpuscular volume of the erythrocytes (MCV), and carbohydrate-deficient-transferrin (CDT).

Direct biomarkers, collected from hair samples, are the following: ethyl glucuronide (EtG; pg/mg) and the sum of the concentrations of four Fatty Acid Ethyl Esters (FAEEs), mainly ethyl myristate (E14:0), ethyl palmitate (E16:0), ethyl stearate (E18:1), and ethyl oleate (E18:0). Body mass index (BMI) was also collected as a potential factor.

**Figure 2:**
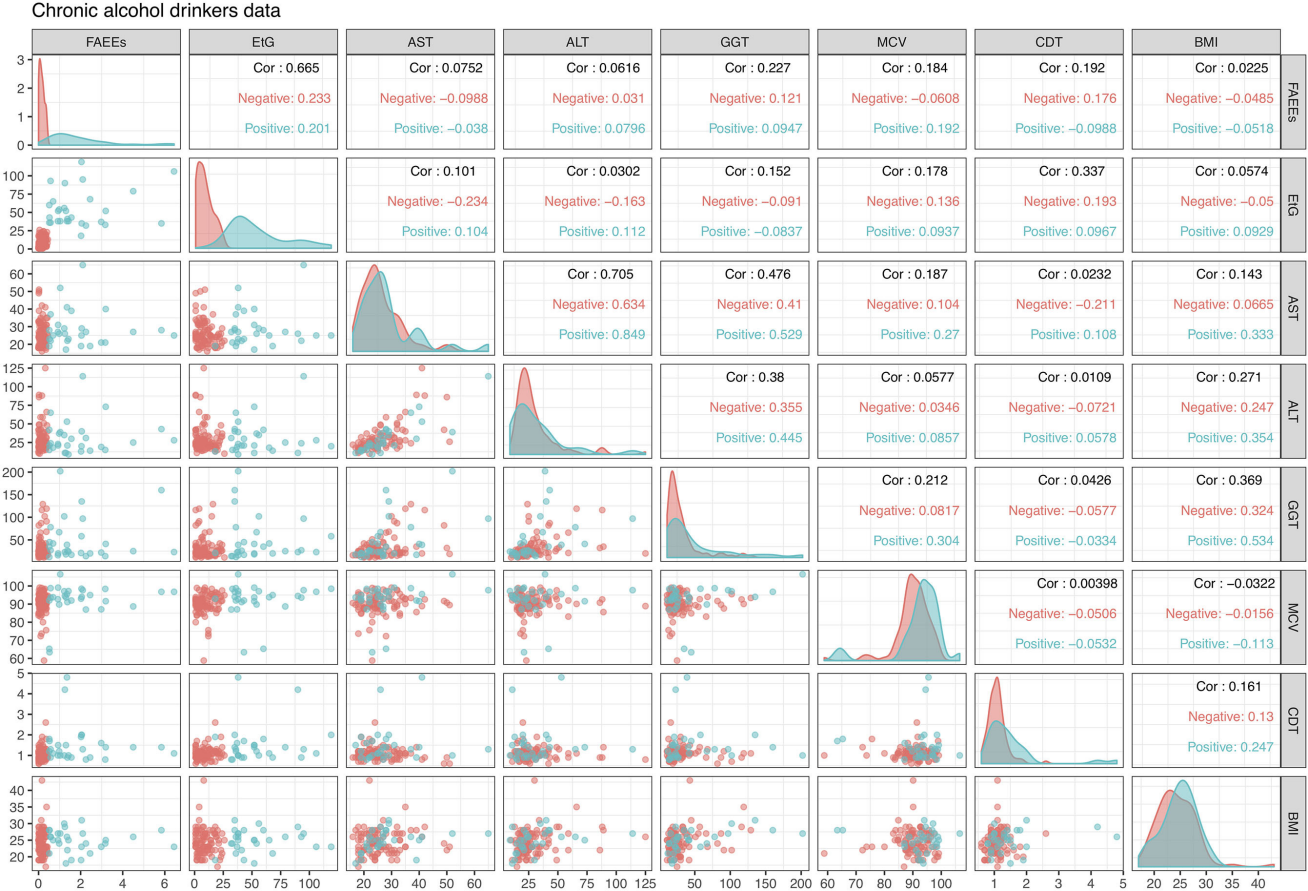
Alcohol biomarkers dataset, with the color indicating the drinking status of the individuals: red is *Negative* (not chronic) and blue is *Positive* (chronic). The upper diagonal panels display the correlation of variables for the whole data but also separately for each class. Diagonal panels show densities of the data by class, while lower diagonal panels show bivariate scatterplots with points colored according to drinking status.

This dataset is displayed in Figure 2 in the form of bivariate scatterplots of these eight variables, where the points are colored according to the alcohol drinking chronic status. Not in particular how in the scatterplot of FAEEs and EtG perfect separation of the positive and negative classes is achieved. While this kind of pattern is often what is needed to achieve a good classification model, this also indicates that a logistic regression model will most likely experience estimation problems and will be unable to provide reliable LR estimates, as discussed in section 2.2.

## 3. Results

In this section we present a comparison of the methods introduced in section 2, using the alcohol biomarkers dataset presented in section 2.8.

Our goal is to demonstrate how separation can be identified and to present alternatives that can be used in practice. These alternatives are penalized logistic regression models, presented in section 2, and can be used both in a classification scenario, using the variables directly, or in a fusion scenario, using the scores of the variables. Lastly, we demonstrate how to run a comparison study when various classification or LR-based methods are available.

### 3.1. Case Study: Alcohol Biomarkers Dataset

In section 2.8, we observe that separation is occurring in the alcohol biomarkers dataset just through visual inspection of plots. However, it is important to know that separation can be more difficult to identify visually when the dataset has a large number of variables and a linear combination of some variables perfectly separates the categories in the data. In this scenario, separation can be detected by inspecting the model estimates for infinite values or any unusual numbers.

For example, a first attempt at fitting a logistic regression model for the alcohol biomarkers dataset fails when using the glm function in R. This is due to the unusually large standard errors, which can be seen in Table 3.

**Table 3:** Logistic regression model coefficients and standard errors, estimated for the alcohol biomarkers dataset, using a logistic regression model implemented in the glm function in R.

	**Intercept**	**FAEEs**	**EtG**	**AST**	**ALT**	**GGT**	**MCV**	**CDT**	**BMI**
Coefficient	−80.353	30.792	2.654	−0.127	−0.197	0.210	0.055	0.246	−0.605
Std. error	2052282.3	75446.6	1899.5	3436.1	2338	1322	30217.2	62386.2	30617.7

*Notice the extremely large standard errors which make it impossible to make further inferences using this model in practice. This model output is to be expected in the presence of separation*.

In contrast, the results from penalized logistic regression models shown in Table 4 indicate that more sensible coefficient estimates and standard errors can be obtained using these methods.

**Table 4:** Coefficients and standard errors, estimated for the alcohol biomarkers dataset, using penalized/Bayesian logistic regression models: Firth GLM ran using the glm function in the R package brglm2, Bayes GLM ran using the bayesglm function in the R package arm and GLM-NET ran using the glmnet function in the R package glmnet (standard errors are not provided by default in the glmnet package; however, they can be estimated using bootstrapping; the dots indicate variables that have been dropped from the model).

	**Intercept**	**FAEEs**	**EtG**	**AST**	**ALT**	**GGT**	**MCV**	**CDT**	**BMI**
**Firth**									
Coefficient	0.171	3.053	0.185	0.118	0.014	−0.020	−0.057	−1.087	−0.162
Std. error	9.015	1.365	0.063	0.087	0.028	0.021	0.076	0.844	0.240
**Bayes GLM**									
Coefficient	−10.581	3.129	0.279	0.010	−0.003	0.004	0.004	0.434	−0.032
Std. error	12.200	1.441	0.101	0.115	0.045	0.027	0.114	1.605	0.22
**GLM-NET**									
Coefficient	−11.191	4.25	0.298	.	.	.	.	.	.

*These methods provide sensible model coefficients and standard errors making them the preferred option for further inferences such as obtaining LRs or prediction for new data*.

The three logistic regression methods discussed in this paper—Firth GLM, Bayes GLM, and GLM-NET—can be used both as classification methods, by using the explanatory variables directly, or within a fusion model (as discussed in section 2.6), where the goal is to combine LRs or scores from different sources.

The rest of this section contains results of a comparison study between the different LR based and classification methods presented in section 2.

As discussed in section 2.7.1, cross-validation should be employed when carrying out a comparison study or when assessing the prediction performance of a model, as this can lead to overly optimistic performance estimates.

For the alcohol biomarkers dataset, which comprises of 125 observations, the dataset is randomly split such that 50% of observations are assigned to the training set, 40% to the validation set and 10% to the testing set. For classification methods, which do not require a multi-step estimation procedure (which happens for LR fusion methods), both the training and validation sets are used to train the classifier, thus ensuring that all the data *not* in the testing set is used in the fitting procedure to train the model. The split and data allocation is repeated *n*_*cv*_ = 50 times, and then averages of the performance measures listed in Table 2 are computed for each algorithm. It should be noted that the results provided using this method are approximate cross-validation scores (since we do not calculate all possible ways to allocate the data), and, due to the Monte Carlo nature of the procedure, the results will be different when the analysis is repeated (unless the random seed generator is saved in advance).

**Figure 3:**
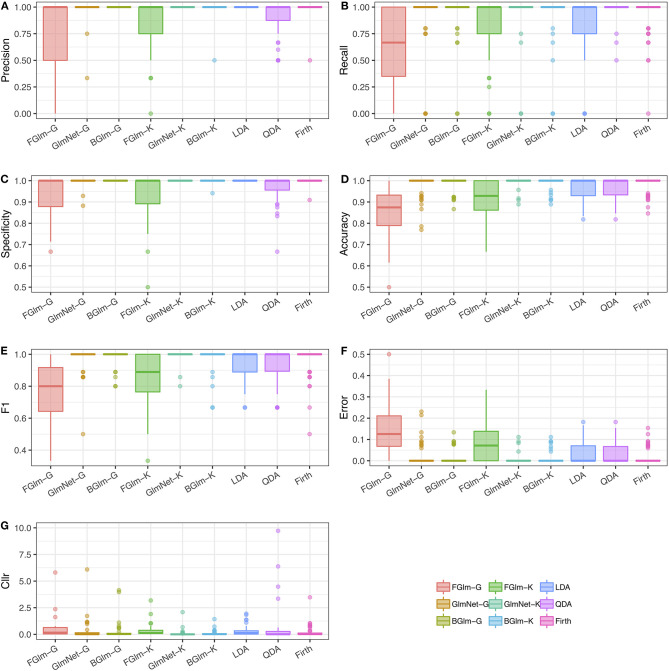
Results of the comparison study between different LR-based and multivariate classification methods. Each figure shows boxplots of different comparison metrics obtained using cross-validation from *n*_*cv*_ = 50 datasets: precision **(A)**, recall **(B)**, specificity **(C)**, accuracy **(D)**, F1 **(E)**, classification error **(F)**, and C_llr_ **(G)** (as defined in Table 2). LR methods using a Gaussian density estimation are indicated with suffix “-G,” while LR methods using KDE are indicated with suffix “-K.” LDA, QDA, and Firth GLM have been included for comparison as classification methods.

The results in Table 5 present cross-validation averages of the various classification performance metrics introduced in section 2.7. On average, all the methods have an accuracy of over 85%, indicating a high probability that individuals will be correctly classified as chronic or non-chronic alcohol drinkers, when the decision-making is based on a threshold of 0.5 for the predicted probability. Figure 3 shows boxplots of all the classification metrics, which gives us an idea of how much variability in the estimates was observed in over the 50 cross-validation datasets. These results indicate that fusion methods based on the Firth GLM model implementation have a larger variability compared to the rest, indicating this method is not as reliable in practice. We experienced some problems with the convergence of the algorithm as implemented in the brglm2 package (Kosmidis, 2020) in four out of the total of 50 dataset in the cross-validation scheme; however, the other implementation of Firth GLM in the R package logistf (Heinze and Ploner, 2018) performed even worse, with more than half of the models returning convergence warnings.

**Table 5:** Classification performance measures for the alcohol biomarkers dataset using penalized Gaussian LR methods, penalized kernel density estimate LR methods and classification methods.

	**Gaussian LR**			**Kernel density estimate LR**			**Classification**		
	**Firth**	**GLM-NET**	**Bayes GLM**	**Firth**	**GLM-NET**	**Bayes GLM**	**LDA**	**QDA**	**Firth**
Precision	0.712	0.980	1	0.838	**1**	0.990	1	0.914	0.990
Recall	0.595	0.897	0.934	0.839	**0.948**	0.934	0.852	0.978	0.920
Specificity	0.934	0.996	1	0.936	**1**	0.999	1	0.967	0.998
Accuracy	0.857	0.975	0.987	0.917	**0.993**	0.989	0.966	0.970	0.981
F1	0.763	0.972	0.984	0.839	**0.993**	0.977	0.943	0.934	0.959
Error	0.143	0.025	0.013	0.083	**0.007**	0.011	0.034	0.030	0.019
C_llr_	1.790	0.293	0.271	0.382	**0.080**	0.098	0.300	0.580	0.244

*Results consist of averages obtained from cross-validation using n_cv_ = 50 datasets and a classification threshold value of 0.5 for the probability (corresponding to 1 for the LR) for obtaining the confusion matrix. Based on the best values across all performance measures (shown as bold values), the best performing method is GLM-NET using KDE estimation*.

The best classification performance, based on the average C_llr_, accuracy, and precision is achieved by the fusion GLM-NET model using kernel density estimation for the scores (see Table 5). Figure 3 shows boxplots of all the classification metrics, which gives us an idea of how much variability in the estimates was observed over the 50 cross-validation datasets. These results indicate that fusion methods based on the Firth GLM model implementation have a larger variability compared to the rest, indicating this method is not as reliable in practice.

GLM-NET with KDE LR estimation has an average precision and specificity rate of 100% meaning no non-chronic individuals get labeled as chronic, when the decision-making is based on a threshold of 0.5 for the predicted probability. Furthermore, the average recall rate is 94.8% which means a chronic individual has, on average, around 5.2% probability to get misclassified as a non-chronic alcohol drinker, when that same probability threshold of 0.5 (equivalent to an LR threshold of 1) is used.

The results for Bayes GLM are very similar to those obtained by GLM-NET, for both Gaussian and KDE LR estimation, while Firth seems to be less reliable—the average C_llr_ is an order of magnitude higher for both of these categories. However, using Firth GLM as a classification algorithm is more stable, with comparable results for precision, recall, accuracy, etc. Interestingly, although the classification methods have a similar performance in terms of metrics based on the confusion matrix, their corresponding C_llr_ values are considerably higher than the best LR methods (GLM-NET and Bayes-GLM using KDE). This suggests that when an observation is misclassified, the LR from these methods gives strong support to the wrong proposition. The C_llr_ thus highlights something that would not be obvious by looking at misclassification error rates alone.

### 3.2. Shiny App

Shiny (Chang et al., 2019) is a package from R software environment (R Core Team, 2019) that allows us to create specific and dynamic interactive web apps. The idea to use R Shiny for developing an open-source tool to perform data analysis came from the necessity to allow analysts, physicians, forensic experts, but also practitioners and laymen in the forensic sciences fields to test, on their own data, several models and statistical approaches aimed to perform robust and comprehensive data evaluations.

We developed an R Shiny app to help practitioners explore the various classification methods discussed in this paper and hopefully apply these well-known algorithms in the statistics and machine learning community to their own datasets.

The app includes functionality for data exploration, classification, and LR-based methods using penalized logistic regression models discussed in this paper.

**Figure 4:**
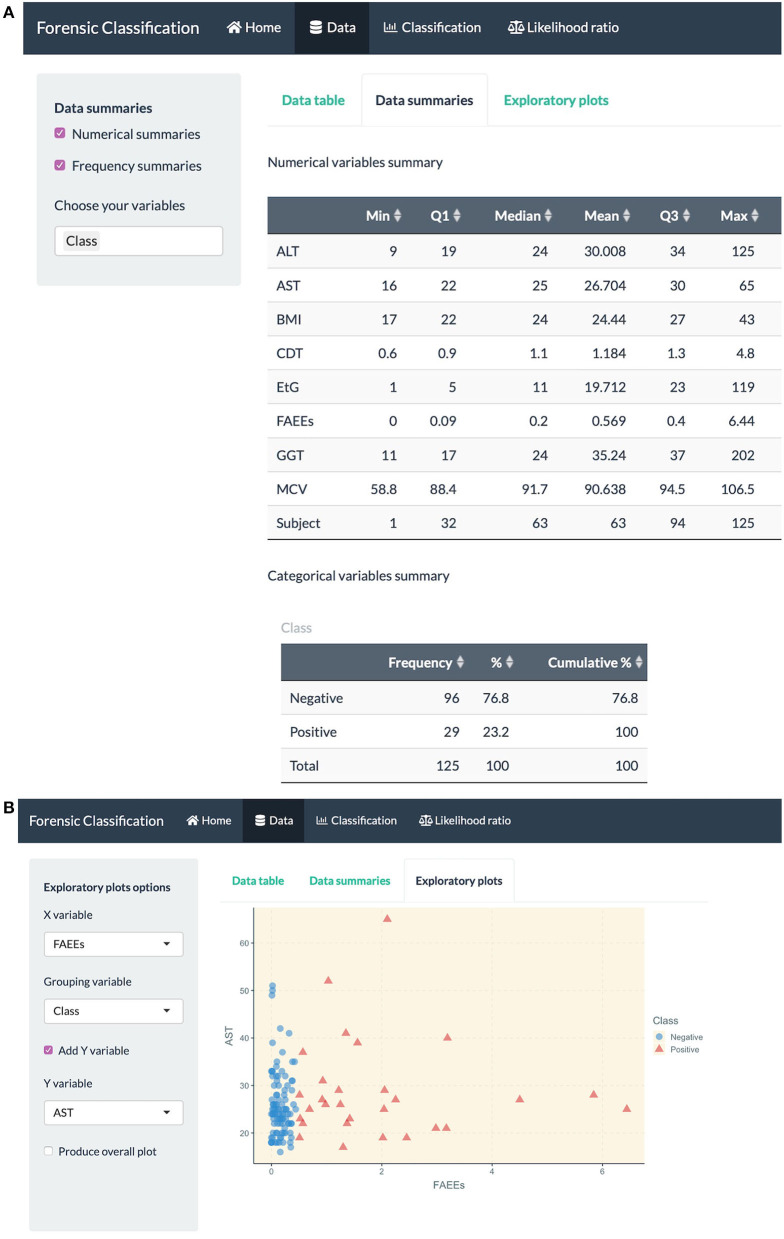
R Shiny app: “Data” tab screenshots, showing **(A)** numerical summaries for variables in the alcohol biomarkers dataset **(B)** exploratory plots.

**Figure 5:**
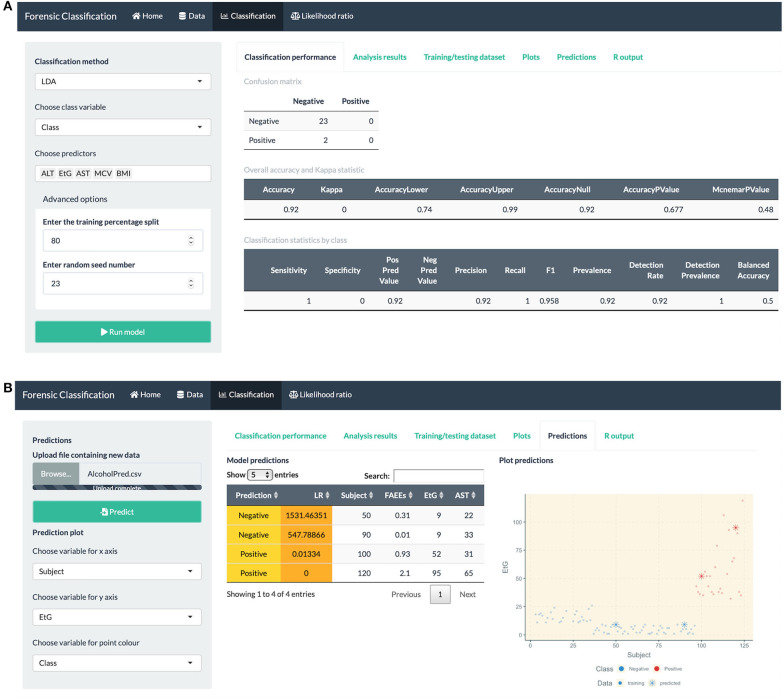
R Shiny app: “Classification” tab screenshots, showing **(A)** confusion matrix and model performance measures: sensitivity, precision, recall, etc. for the alcohol biomarkers dataset **(B)** prediction table, including the predicted class and LR for each observation, and plot showing the training and predicted data.

**Figure 6:**
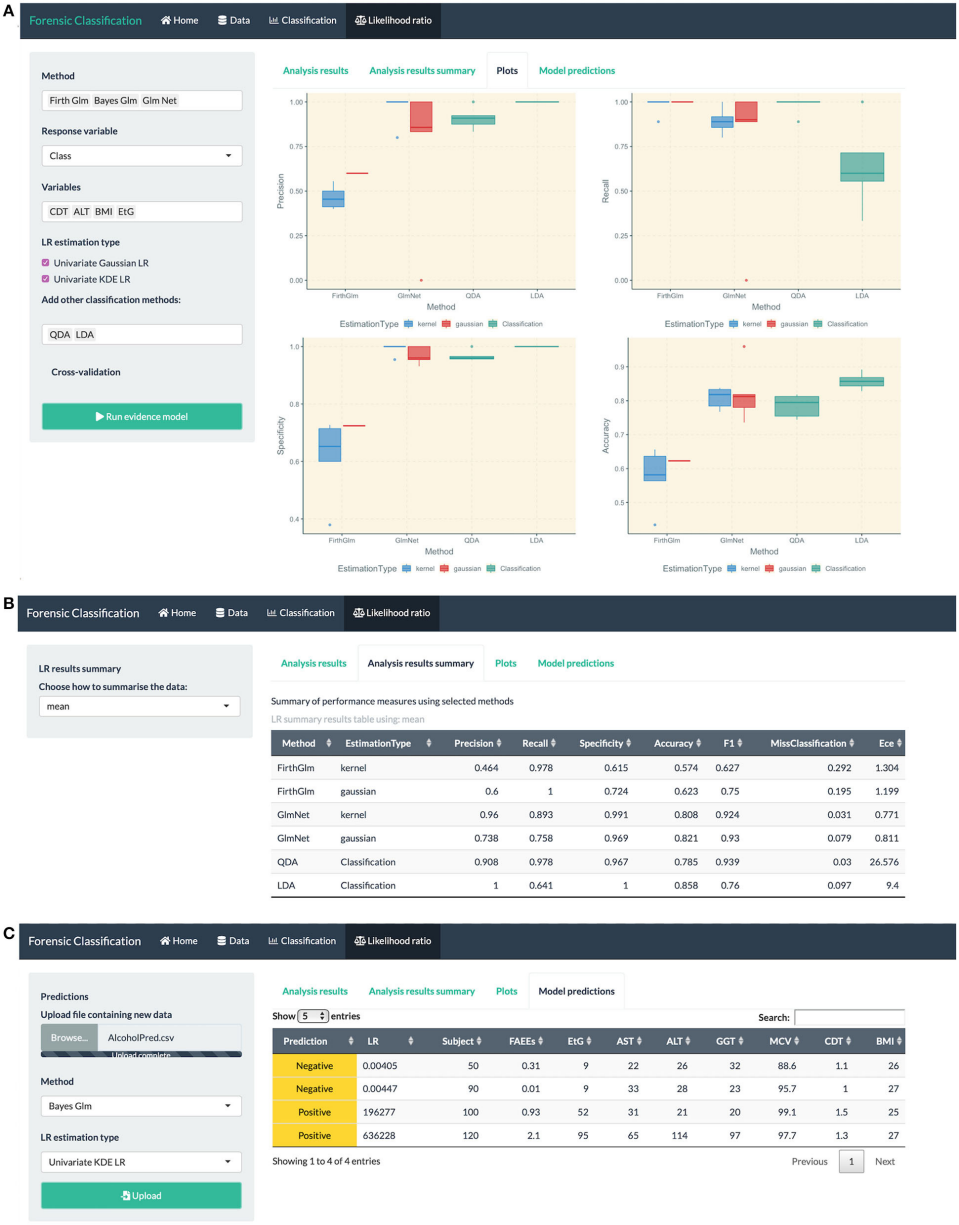
R Shiny app: “Likelihood ratio” tab screenshots, showing in **(A)** model comparison based on LR combination methods (Firth and GLM-NET) and regular classification methods such as LDA and QDA, in **(B)** summary of performance measures for method comparison, in **(C)** prediction table based on the Bayes GLM method with scores estimated using KDE, including the predicted class and LR for each observation in the uploaded dataset.

Here are some of the R Shiny app capabilities in more detail:

- data exploration through numerical and visual summaries, see Figure 4.
- fitting classification methods for bivariate and multiclass datasets, such as LDA, QDA, logistic regression, Firth GLM, and multinomial logistic regression, model summaries, classification performance measures and visualization plots, see Figure 5.
- fitting LR combination methods using penalized logistic regression models, such as GLM-NET, Firth or Bayes GLM, and LR estimation based on a Gaussian distribution or KDE, see Figure 6 (top).
- method comparison using cross-validation based on metrics discussed in this paper, see Figure 6 (middle).
- the user can explore and gain an understanding of these methods using in-built datasets which are routinely used in the machine learning community for classification and prediction.
- uploading an external dataset to run the classification or LR combination methods, see Figure 6 (bottom).
- prediction capabilities for external data that can be uploaded into the app, see Figure 5 (bottom).

Additional datasets available within the app are:

*Iris* (Dua and Graff, 2019) Contains data from three species of iris: setosa, versicolor, and virginica. There are four measurements collected that have been included in this dataset: the sepal length, sepal width, petal length, and petal width in cm. The number of observations in each class is balanced (50 observations per class, 150 in total). It is a multi-class dataset with 150 observations, four explanatory variables, and one output variable.
*Glass* (Dua and Graff, 2019) It contains data representing seven types of glass: building windows (float processed), building windows (non-float processed), vehicle windows (float processed), vehicle windows (non-float processed), containers, tableware, and headlamps. There are nine explanatory variables: refractive index, along with the chemical composition measured as weight percent in corresponding oxide, Sodium, Magnesium, Aluminum, Silicon, Potassium, Calcium, Barium, and Iron. The dataset is imbalanced, meaning that the 214 total observations are not distributed equally across the seven classes.
*Diabetes* (Dua and Graff, 2019) This dataset contains measurements of 768 females over 21 years old of Pima Indian heritage, collected with the goal of predicting whether a patient has diabetes (this represents the class variable). The variables collected are: the number of times pregnant, plasma glucose concentration a 2 h in an oral glucose tolerance test, diastolic blood pressure, triceps skin fold thickness, 2-h serum insulin, body mass index, age, and the diabetes pedigree function.
*Diamonds* (Wickham, 2016) Contains 10 variables recorded for different types of diamonds: price, carat, length, width, depth, total depth percentage, and the width of the top of the diamond relative to the widest point. There are three categorical variables in this dataset describing the quality of the cut (Fair, Good, Very Good, Premium, and Ideal), the diamond color (from J–worst to D–best), and the clarity (I1–worst, SI2, SI1, VS2, VS1, VVS2, VVS1, and IF–best). There are 53,940 observations in this dataset.

The Shiny app is free to use and can be found at the following link: https://dianagiurghita.shinyapps.io/ForensicClassification/

## 4. Discussion

LR-based methods, such as logistic regression fusion (Morrison, 2011b, 2013) and classification methods such as LDA and QDA are attractive to forensic experts because they allow them to carry out a rigorous, sound statistical analysis. Furthermore, these methods allow experts to present to the courtroom a likelihood ratio, which can easily be put into a statement to convey the strength of the evidence obtained from their analysis for various hypotheses of interest in a case.

In this paper, we present a framework for classification based on penalized logistic regression methods: Firth GLM, Bayes GLM, and GLM-NET. These algorithms are widely known and used in the statistics and machine learning communities as algorithms that can accommodate a large number of explanatory variables, sparse datasets, and correlated variables and can deal with separation or quasi-separation in the data; however, they seem to be less known outside these circles. These methods should be regarded as an extension to logistic regression and logistic regression fusion since they accomplish the same role—they perform classification, prediction, and return LRs—but they have built-in mechanisms to deal with some common estimation problems.

Another extension we provide in this paper is in the context of fusion of LRs: to this end, we present kernel density estimation as an alternative to the widely used Gaussian distribution approximation. This is suitable in situations when the underlying data is multi-modal or not particularly symmetric or bell-shaped.

We demonstrate the use of these penalized logistic regression algorithms on an alcohol biomarkers dataset which includes direct and indirect biomarkers for the identification of chronic alcohol drinkers. These two categories of alcohol drinkers (chronic and non-chronic) can be perfectly separated by two variables in the dataset, and we indicate how to recognize that estimation fails in such scenarios using a logistic regression model. Furthermore, we present a comparison study using the penalized logistic regression model framework proposed in section 2. The best model based on C_llr_, accuracy and precision is GLM-NET using KDE estimation for LRs, with Bayes-GLM providing similar performance. We find that models based on Firth GLM are less reliable and this can be due to a mixture of factors, including the software implementation being unstable and the choice of penalty involving a prior that is too weak (as pointed out in Gelman et al., 2008).

Lastly, we hope to encourage practitioners to learn more about these methods and apply them when necessary and, to this end, we have built a user-friendly R Shiny app that is freely available and very comprehensive. The app includes all the methods presented in this paper and has in-built datasets that allow users to explore and get a better understanding of penalized logistic regression models.

## Data Availability Statement

The alcohol biomarkers dataset used in this paper is published in Alladio et al. (2017a). The R Shiny app can be downloaded at: https://github.com/DianaGiurghita/Forensic-Classification or used online at: https://dianagiurghita.shinyapps.io/ForensicClassification/.

## Author Contributions

EA, GB, and TN devised the project and the main conceptual ideas. GB, EA, and MV designed and implemented the study that produced the alcohol biomarkers dataset. DG and TN designed the R Shiny app, researched the appropriate statistical methodology, and carried out the data analysis. EA and GB provided feedback and tested the R Shiny app. DG, GB, and EA wrote the manuscript. All authors read and approved the submitted version.

## Conflict of Interest

The authors declare that the research was conducted in the absence of any commercial or financial relationships that could be construed as a potential conflict of interest.

## References

[B1] Aitken C. Taroni F. (2004). Statistics and the Evaluation of Evidence for Forensic Scientists. Chichester: John Wiley & Sons 10.1002/0470011238

[B2] Alladio E. Giacomelli L. Biosa G. Di Corcia D. Gerace E. Salomone A.. (2018). Development and validation of a Partial Least Squares-Discriminant Analysis (PLS-DA) model based on the determination of ethyl glucuronide (EtG) and fatty acid ethyl esters (FAEEs) in hair for the diagnosis of chronic alcohol abuse. Forens. Sci. Int. 282, 221–230. 10.1016/j.forsciint.2017.11.010 29174052

[B3] Alladio E. Martyna A. Salomone A. Pirro V. Vincenti M. Zadora G. (2017a). Direct and indirect alcohol biomarkers data collected in hair samples-multivariate data analysis and likelihood ratio interpretation perspectives. 12, 1–8. 10.1016/j.dib.2017.03.026 28607948 PMC5457474

[B4] Alladio E. Martyna A. Salomone A. Pirro V. Vincenti M. Zadora G. (2017b). Evaluation of direct and indirect ethanol biomarkers using a likelihood ratio approach to identify chronic alcohol abusers for forensic purposes. Forens. Sci. Int. 70, 13–22. 10.1016/j.forsciint.2016.12.019 28056375

[B5] Bishop C. M. (2006). Pattern Recognition and Machine Learning. New York, NY: Springer.

[B6] Chang W. Cheng J. Allaire J. Xie Y. McPherson J. (2019). shiny: Web Application Framework for R. R Package Version 1.4.0.

[B7] Dua D. Graff C. (2019). UCI Machine Learning Repository. Irvine, CA: University of California, School of Information and Computer Sciences Available online at: http://archive.ics.uci.edu/ml (accessed October 15, 2019).

[B8] European Network of Forensic Science Institutes (2016). ENFSI Guideline for Evaluative Reporting in Forensic Science. Dua, D., and Graff, C. (2017). UCI Machine Learning Repository. Irvine, CA: University of California, Irvine, School of Information and Computer Sciences Available online at: http://archive.ics.uci.edu/ml (accessed October 15, 2019).

[B9] Evett I. Jackson G. Lambert J. McCrossan S. (2000). The impact of the principles of evidence interpretation on the structure and content of statements. Sci. Justice 40, 233–239. 10.1016/S1355-0306(00)71993-9 11094820

[B10] Evett I. W. Weir B. S. (1998). Interpreting DNA Evidence: Statistical Genetics for Forensic Scientists, Vol. 244. Sunderland, MA: Sinauer Associates.

[B11] Firth D. (1993). Bias reduction of maximum likelihood estimates. Biometrika 80, 27–38. 10.1093/biomet/80.1.27

[B12] Friedman J. Hastie T. Tibshirani R. (2001). The Elements of Statistical Learning, Vol. 1. New York, NY: Springer 10.1007/978-0-387-21606-5_1

[B13] Friedman J. Hastie T. Tibshirani R. (2010). Regularization paths for generalized linear models via coordinate descent. J. Stat. Softw. 33, 1–22. 10.18637/jss.v033.i01 20808728 PMC2929880

[B14] Gelman A. Carlin J. B. Stern H. S. Dunson D. B. Vehtari A. Rubin D. B. (2013). Bayesian Data Analysis. Boca Raton, FL: CRC Press 10.1201/b16018

[B15] Gelman A. Jakulin A. Pittau M. G. Su Y. (2008). A default prior distribution for logistic and other regression models. Ann. Appl. Stat. 2, 1360–1383. 10.1214/08-AOAS191

[B16] Gelman A. Su Y.-S. Yajima M. Hill J. Pittau M. G. Kerman J. (2018). Package Arm. R Package Version 1.10-1.

[B17] Gill P. Brenner C. Buckleton J. Carracedo A. Krawczak M. Mayr W.. (2006). DNA commission of the international society of forensic genetics: recommendations on the interpretation of mixtures. Forens. Sci. Int. 160, 90–101. 10.1016/j.forsciint.2006.04.009 16750605

[B18] Gonzalez-Rodriguez J. Rose P. Ramos D. Toledano D. T. Ortega-Garcia J. (2007). Emulating DNA: Rigorous quantification of evidential weight in transparent and testable forensic speaker recognition. IEEE Trans. Audio Speech Lang. Process. 15, 2104–2115. 10.1109/TASL.2007.902747

[B19] Hastie T. Qian J. (2014). Glmnet Vignette. Available online at: http://www. web. stanford. edu/ hastie/Papers/Glmnet_Vignette. pdf (accessed September 20, 2016).

[B20] Heinze G. Ploner M. (2018). logistf: Firth's Bias-Reduced Logistic Regression. R Package Version 1.23.

[B21] Heinze G. Schemper M. (2002). A solution to the problem of separation in logistic regression. Stat. Med. 21, 2409–2419. 10.1002/sim.1047 12210625

[B22] Kintz P. Salomone A. Vincenti M. (2015). Hair Analysis in Clinical and Forensic Toxicology. Amsterdam: Academic Press.

[B23] Kosmidis I. (2020). brglm2: Bias Reduction in Generalized Linear Models. R Package Version 0.6.2.

[B24] Kosmidis I. Pagui E. C. K. Sartori N. (2020). Mean and median bias reduction in generalized linear models. Stat. Comput. 30, 43–59. 10.1007/s11222-019-09860-6

[B25] Lucy D. (2013). Comparison: Multivariate Likelihood Ratio Calculation and Evaluation. R Package Version 1.0-4.

[B26] Mai Q. (2013). A review of discriminant analysis in high dimensions. Wiley Interdisc. Rev. 5, 190–197. 10.1002/wics.1257

[B27] Martyna A. Michalska A. Zadora G. (2015). Interpretation of FTIR spectra of polymers and Raman spectra of car paints by means of likelihood ratio approach supported by wavelet transform for reducing data dimensionality. Analyt. Bioanalyt. Chem. 407, 3357–3376. 10.1007/s00216-015-8558-9 25757825

[B28] Michalska A. Martyna A. Zieba-Palus J. Zadora G. (2015). Application of a likelihood ratio approach in solving a comparison problem of Raman spectra recorded for blue automotive paints. J. Raman Spectrosc. 46, 772–783. 10.1002/jrs.4719

[B29] Morrison G. S. (2011a). A comparison of procedures for the calculation of forensic likelihood ratios from acoustic-phonetic data: multivariate kernel density (MVKD) versus Gaussian mixture model-universal background model (GMM-UBM). Speech Commun. 53, 242–256. 10.1016/j.specom.2010.09.005

[B30] Morrison G. S. (2011b). Measuring the validity and reliability of forensic likelihood-ratio systems. Sci. Just. 51, 91–98. 10.1016/j.scijus.2011.03.002 21889105

[B31] Morrison G. S. (2013). Tutorial on logistic-regression calibration and fusion: converting a score to a likelihood ratio. Austr. J. Forens. Sci. 45, 173–197. 10.1080/00450618.2012.733025

[B32] Morrison G. S. Poh N. (2018). Avoiding overstating the strength of forensic evidence: shrunk likelihood ratios/Bayes factors. Sci. Just. 58, 200–218. 10.1016/j.scijus.2017.12.005 29685302

[B33] Murphy K. P. (2012). Machine Learning: A Probabilistic Perspective. Cambridge, MA: MIT Press.

[B34] Pirro V. Oliveri P. Sciutteri B. Salvo R. Salomone A. Lanteri S.. (2013). Multivariate strategies for screening evaluation of harmful drinking. Bioanalysis 5, 687–699. 10.4155/bio.13.12 23484786

[B35] Pragst F. Rothe M. Moench B. Hastedt M. Herre Simmert D. (2010). Combined use of fatty acid ethyl esters and ethyl glucuronide in hair for diagnosis of alcohol abuse: interpretation and advantages. Forens. Sci. Int. 196, 101–110. 10.1016/j.forsciint.2009.12.028 20061103

[B36] Qin Y. (2018). A review of quadratic discriminant analysis for high-dimensional data. Wiley Interdisc. Rev. 10:e1434 10.1002/wics.1434

[B37] R Core Team (2019). R: A Language and Environment for Statistical Computing. Vienna: R Foundation for Statistical Computing.

[B38] Ramos D. (2007). Forensic evaluation of the evidence using automatic speaker recognition systems (Ph.D. thesis). Universidad autónoma de Madrid, Madrid, Spain.

[B39] Ramos D. Franco-Pedroso J. Lozano-Diez A. Gonzalez-Rodriguez J. (2018). Deconstructing cross-entropy for probabilistic binary classifiers. Entropy 20:208 10.3390/e20030208 PMC7512723 33265299

[B40] Robertson B. Vignaux G. A. Berger C. E. (2016). Interpreting Evidence: Evaluating Forensic Science in the Courtroom. Chichester: John Wiley & Sons 10.1002/9781118492475

[B41] Silverman B. (1986). Density Estimation for Statistics and Data Analysis. London: Chapman and Hall 10.1007/978-1-4899-3324-9

[B42] Society of Hair Testing (2019). 2019 Consensus for the Use of Alcohol Markers in Hair for Supporting the Assessment of Abstinence and Chronic Alcohol Consumption.

[B43] Venables W. N. Ripley B. D. (2013). Modern Applied Statistics With S-PLUS. New York, NY: Springer Science & Business Media.

[B44] Wasserstein R. L. Lazar N. A. (2016). The ASA statement on p-values: context, process, and purpose. Am. Stat. 70, 129–133. 10.1080/00031305.2016.1154108

[B45] Wickham H. (2016). ggplot2: Elegant Graphics for Data Analysis. New York, NY: Springer-Verlag Available online at: https://ggplot2.tidyverse.org (accessed October 15, 2019).

[B46] Zadora G. (2010). Evaluation of evidential value of physicochemical data by a Basyesian network approach. J. Chemometr. 24, 346–366. 10.1002/cem.1307

[B47] Zadora G. Borusiewicz R. Zieba-Palus J. (2005). Differentiation between weathered kerosene and diesel fuel using automatic thermal desorption-GC-MS analysis and the likelihood ratio approach. J. Separ. Sci. 28, 1467–1475. 10.1002/jssc.200400085

[B48] Zadora G. Martyna A. Ramos D. Aitken C. (2014). Statistical Analysis in Forensic Science - Evidential Value of Multivariate Physicochemical Data, 1st Edn. Chicester: John Wiley & Sons, Ltd 10.1002/9781118763155

[B49] Zadora G. Ramos D. (2010). Evaluation of glass samples for forensic purposes - an application of likelihood ratios and an information theoretical approach. Chemometr. Intell. Lab. Syst. 8.2, 63–83. 10.1016/j.chemolab.2010.03.007

